# Periodontal Status and Oral Health–Related Quality of Life in Patients With Reticular and Nonreticular Oral Lichen Planus: A Cross‐Sectional Study

**DOI:** 10.1002/cre2.70390

**Published:** 2026-06-16

**Authors:** Linda Daume, Sonja Sielker, Ole Oelerich, Johannes Kleinheinz, Lauren Bohner

**Affiliations:** ^1^ Department of Cranio‐Maxillofacial Surgery University Hospital Muenster Muenster Germany

**Keywords:** OHIP‐14, oral health, oral lichen planus, PSI

## Abstract

**Background:**

Oral lichen planus (OLP) is a chronic inflammatory mucocutaneous disease that may affect oral health parameters. This study examined the relationships between periodontal status, dental health, and oral health‐related quality of life (OHRQoL) in patients with reticular and nonreticular OLP.

**Methods:**

This cross‐sectional observational study included 112 patients from the Department of Cranio‐Maxillofacial Surgery with histopathologically confirmed OLP. Periodontal status was assessed using the periodontal screening index (PSI), dental health was assessed using the decayed, missing, filled teeth (DMFT) index, and OHRQoL was assessed using the German Oral Health Impact Profile 14 (OHIP‐14). Patients were stratified into reticular and nonreticular OLP groups. Analyses were exploratory and based on group comparisons and correlation statistics.

**Results:**

Patients with nonreticular OLP demonstrated significantly higher PSI scores than those with reticular OLP (*p* < 0.01), indicating poorer periodontal health. DMFT scores did not differ between groups. OHRQoL was significantly impaired in patients with nonreticular OLP (OHIP‐14: 15.55 ± 10.60 vs. 11.06 ± 10.88; *p* = 0.02). The OHIP‐14 showed a moderate association with the PSI and a weak association with the DMFT.

**Conclusions:**

The clinical subtype of OLP is associated with differences in periodontal status and OHRQoL. Further longitudinal studies are required. These findings suggest that oral health conditions may be worth considering in the clinical management of OLP patients.

**Trial Registration:**

The study was prospectively registered (Ref. No. 2019‐033.f‐S) in accordance with institutional ethical requirements.

## 1 Introduction

Oral lichen planus (OLP) is a chronic inflammatory mucocutaneous disease of unknown etiology, although evidence suggests an immunomodulated mechanism (Gupta and Jawanda 2015; Sugerman et al. 2002). In 1978, the WHO developed a definition of OLP incorporating clinical and histopathological aspects (Thongprasom et al. 2011). OLP has a global prevalence ranging from 0.05% to 3.25%, with a higher incidence in females, peaking in the fourth and fifth decades of life (Andreasen 1968; Al‐Hashimi et al. 2007). Malignant transformation occurs at a rate of 1.2% (Salgado et al. 2013).

Oral lesions frequently occur in a bilateral and symmetrical distribution. The most common site is the buccal mucosa, followed by the gingiva and tongue (Thongprasom et al. 2011).

According to Andreasen's classification, there are six forms of OLP: reticular, papular, plaque, atrophic, erosive‐ulcerative, and bullous. Reticular OLP is the most common form (Andreasen 1968). Symptoms range from mild discomfort to intense pain, particularly in the atrophic, erosive, and bullous forms.

Currently, there is no definitive cure for OLP; treatment focuses on symptom management, primarily using topical corticosteroids due to their effectiveness and minimal side effects (Al‐Hashimi et al. 2007).

In addition to the topical treatment of OLP, any mechanical injuries or existing local irritants, such as poorly fitting restorations, must be eliminated. Furthermore, establishing an effective oral hygiene program is crucial, particularly for patients with gingival involvement. Poor oral health conditions, such as dental biofilms and periodontal disease, can influence the clinical development of OLP lesions, and these conditions are likely to exacerbate and maintain the inflammatory process at the lesion site. It has been demonstrated that plaque control improves the symptoms of gingival OLP (Salgado et al. 2013). According to some authors, metal and amalgam restorations may worsen or even cause OLP (Ismail et al. 2007). However, the evidence regarding periodontal status of patients with OLP remains limited and heterogeneous, and standardized assessments using validated periodontal indices are scarce.

Several studies have suggested an association between OLP and impaired oral health parameters, including increased plaque levels and altered periodontal conditions. Nevertheless, these studies are often limited by small sample sizes, inconsistent diagnostic criteria, or a lack of differentiation between clinical OLP subtypes. Moreover, the impact of periodontal status on patient‐reported outcomes, such as oral health‐related quality of life (OHRQoL), particularly in relation to the different clinical forms of OLP, has not been adequately investigated.

Beyond mucosal involvement, OLP may also influence overall oral health. Due to its chronic nature, OLP significantly affects patients’ oral function, social interactions, and psychological well‐being (Liu et al. 2012). Anxiety, depression, and stress have been suggested as potential triggers for OLP manifestations (Gavic et al. 2014; Dalirsani et al. 2011). A review of OLP confirmed its substantial negative impact on OHRQoL, particularly in patients with erosive or bullous OLP compared to those with reticular forms (Cheng et al. 2016; Parlatescu et al. 2020). However, the combined relationship between periodontal status, dental health parameters, and OHRQoL across different clinical presentations of OLP remains unclear.

Therefore, the aim of this cross‐sectional observational study was to investigate the association between periodontal status (assessed using the periodontal screening index, PSI), dental health (assessed using the decayed, missing, filled teeth [DMFT] index), and oral health–related quality of life (assessed using the OHIP‐14 questionnaire) in patients with reticular and nonreticular OLP. This exploratory study does not aim to test a causal hypothesis, but rather to identify associations between oral health conditions and patient‐reported outcomes across different clinical forms of OLP.

## 2 Materials and Methods

This prospective, cross‐sectional, observational study included 112 patients diagnosed with OLP at the Department of Cranio‐Maxillofacial Surgery, a tertiary care center. The single‐center design may introduce potential selection bias, a limitation acknowledged in the study.

The study was approved by the Ethics Committee (Ref. No. 2019‐033.f‐S). Written informed consent was obtained from all participants. All procedures were conducted in accordance with the Declaration of Helsinki.

Patients were included based on clinical and histopathological confirmation of OLP using modified WHO diagnostic criteria (Kramer et al. 1978). All examinations were performed under standardized clinical conditions by an experienced and trained oral surgeon. Although examiner calibration was ensured through standardized clinical training, formal inter‐examiner reliability testing was not performed, which is recognized as a methodological limitation.

Inclusion criteria were a minimum age of 18 years and good knowledge in spoken and written German. Exclusion criteria included the presence of other oral mucosal diseases or oral lichenoid lesions, and prior head and neck radiotherapy.

A clinical dental examination was performed using a dental mirror and probe under artificial light in the treatment room. The PSI is an element of the dental examination (Meyle and Jepsen 2000). While the PSI allows a standardized estimation of periodontal treatment need, it does not represent a full periodontal charting and therefore only provides a screening‐level assessment of periodontal health. The PSI was performed using a WHO periodontal probe in all sextants. The probe was gently inserted between the tooth and the gum at six different sites on each tooth (mesiobuccal, buccal, distobuccal, mesiolingual, lingual, and distobuccal), and the highest value for each sextant was recorded. In addition to the probing depth, bleeding on probing (BoP) and any existing roughness or deposits on the tooth surface were determined. Diagnosis and treatment implications were then determined according to the PSI code, as determined by Rukat and Kielbassa (2004) (Table 1).

**Table 1:** PSI codes and their implications (Rukat and Kielbassa 2004).

PSI codes		
Diagnosis	Code	Implications
Healthy gingiva; the black band of the WHO probe remains completely visible	0	No further therapy necessary; preventive care
Bleeding after careful probing; the black band remains completely visible; no tartar; no defective restoration margins	1	Gingivitis: plaque removal and oral hygiene instruction required
Bleeding after probing; the black band remains completely visible; tartar and/or defective restoration margins	2	Gingivitis: improvement of oral hygiene, PTC and elimination of other "irritating factors" necessary
The black band remains partially visible at the deepest of the sulcus of all teeth of a sextant	3	Moderate periodontitis: improvement of oral hygiene, further diagnostic and therapeutic measures, PTC, increased need for treatment (if necessary, in the entire dentition)

The number of decayed, missing (for any reason and excluding third molars), and filled teeth (DMFT) was recorded. The DMFT index is recommended by the WHO for assessing the number of decayed, missing, and filled teeth (John et al. 2006). To determine the index, patients were clinically examined. The maximum score was set at 28, and the minimum score was set at 0. For statistical purposes, the overall score was considered. We classified the indices into three groups (group 1: 0–10; group 2: 11–20; and group 3: 21–28). This categorization was applied for descriptive stratification; however, it may reduce variability and statistical power, a limitation that is acknowledged.

All patients completed the German version of the short form of the Oral Health Impact Profile (OHIP‐14) questionnaire. The 14 items, relating to seven conceptual domains (“functional limitation,” “physical pain,” “psychological discomfort,” “physical disability,” “psychological disability,” “social disability,” and “handicap”) were evaluated using a five‐point scale. The possible responses were 0 indicating “never,” 1 indicating “hardly ever,” 2 indicating “occasionally,” 3 indicating “fairly often,” and 4 indicating “very often.” For statistical analysis, the overall additive score (the primary outcome, ranging from 0 to 56) was used, with higher scores indicating poorer OHRQoL. Additional patients were asked yes/no questions about their oral health (dental check‐ups, professional teeth cleaning (PTC), gingival bleeding, and perceived plaque accumulation).

Potential confounding variables such as smoking status, systemic diseases, medication intake, and socioeconomic factors were not systematically recorded or adjusted for in the statistical analysis. This is acknowledged as a limitation of the study.

### 2.1 Statistical Analysis

SPSS software version 28.0 (IBM Group Armonk, NY: IBM Corp.) was used for statistical analysis. The chi‐square test was used to analyze categorical data. OHIP‐14 scores were assessed using the Mann–Whitney *U* test. Spearman's rho correlation coefficient was used to investigate correlations between OHIP‐14 scores and the OLP form, oral health conditions (DMFT) and PSI. The degree of correlation was graded as follows: 0.2–0.5 indicates a low correlation, 0.5–0.7 indicates a moderate correlation, 0.7–0.9 indicates a good correlation, and 0.9–1.0 indicates a high correlation (Krentz 2005). As no multivariable regression models were applied, the results represent unadjusted associations and should be interpreted as exploratory. Statistical significance was set at *p* < 0.05.

## 3 Results

A total of 112 people (21 men and 91 women) with clinically and histopathologically confirmed OLP participated in this study. The mean age was 59.98 ± 10.69 years (range 26.6–85.3 years) (Table 2). The most common clinical form was the reticular form (group 1). Patients presenting reticular white lesions without atrophy, erosion, ulceration or papular‐ or plaque‐type lesions were assigned to this group. The erosive‐ulcerative, bullous and atrophic forms were summarized in group 2 (nonreticular OLP). Papular and plaque‐type OLP were not present in our group of patients.

**Table 2:** Demographic data, clinical parameters, and survey results of participants with OLP.

Variables	Total (*n* = 112)	Reticular OLP (*n* = 50)	Nonreticular^a^ OLP (*n* = 62)	*p* value
Gender (%)				
Female	91 (81.25)	38 (76)	53 (84.48)	0.20
Male	21 (18.75)	12 (24)	9 (14.52)	
PSI (%)				
0	0 (0)	0 (0)	0 (0)	0.006 **< 0.01**
1	7 (6.3)	5 (10)	2 (3.2)	
2	36 (32.1)	23 (46)	13 (21)	
3	49 (43.8)	17 (34)	32 (51.6)	
4	20 (17.9)	5 (10)	15 (24.2)	
DMFT (%)				
1	8 (7.1)	3 (6)	5 (8.06)	0.45
2	62 (55.4)	31 (62)	31 (50)	
3	42 (37.5)	16 (32)	26 (41.94)	
Dental check‐up (%)				
Dental check‐up	107 (95.5)	2 (4)	3 (4.6)	0.83
No dental check‐up	5 (4.5)	48 (96)	59 (95.2)	
PTC (%)				
Regular	85 (75.9)	39 (78)	46 (74.2)	0.64
Not regular	27 (24.1)	11 (22)	16 (25.8)	
Bleeding gums (%)				
Bleeding	41 (36.6)	13 (26)	28 (45.2)	**0.036**
No bleeding	71 (63.4)	37 (74)	34 (54.8)	
Plaque				
Plaque (%)	58 (51.8)	25 (50)	33 (46.8)	0.73
No plaque (%)	54 (48.2)	25 (50)	29 (53.2)	

*Note:* Bold values are statistically significant.

^a^ Erosive‐ulcerative OLP, bullous OLP, and atrophic OLP.

The reticular form (group 1) was diagnosed in 44.64% (*n* = 50) of patients. The erosive‐ulcerative, bullous, and atrophic forms (group 2) were found in 55.36% (*n* = 62) of patients. There was no difference in gender distribution between the two groups (Table 2).

Periodontal screening revealed that 61.7% of the patients examined had periodontitis (PSI codes 3 and 4). Patients with nonreticular OLP had higher PSI scores than those with reticular OLP, indicating worse periodontal conditions. This difference was statistically significant (*p* < 0.01).

In general, patients had a mean DMFT value of 18.02 ± 5.59 (range 3–28), indicating a generally moderate level of cumulative dental disease burden. The DMFT values did not differ between patients with reticular and nonreticular OLP, suggesting comparable long‐term caries experience across clinical subtypes (Table 2).

A total of 95.5% of all participants had regular dental check‐ups, and 75.9% received a PTC. No meaningful differences in preventive care behaviors were observed between the two OLP subgroups.

Self‐reported gingival bleeding was reported by 36.6% of patients overall, being more frequent in the nonreticular group. This difference was statistically significant (*p* = 0.036) (Table 2), indicating a higher burden of inflammatory symptoms in this subgroup. Reports of plaque presence were common (51.8%), but did not differ meaningfully between groups and showed no clear association with clinical OLP subtype.

The average total OHIP‐14 score for the entire cohort was 13.54 ± 10.91, indicating a moderate impairment of OHRQoL. Nonreticular OLP patients reported significantly higher OHIP‐14 scores (15.55 ± 10.6) than reticular OLP patients (*p* = 0.02) (Table 3). This difference suggests a clinically relevant reduction in OHRQoL in patients with a more symptomatic form of OLP.

**Table 3:** OHIP‐14 according to the clinical form of disease.

	Total (*n* = 112)		Reticular (*n* = 50)		Nonreticular^a^ (*n* = 62)		
Variables	Mean (± SD)	Median IQR	Mean (± SD)	Median IQR	Mean (± SD)	Median IQR	*p* value*
OHIP‐14	13.54 ± 10.91	11 (0–45)	11.06 ± 10.88	16 (0–45)	15.55 ±10.6	7 (0–43)	**0.02** *

^a^ Erosive‐ulcerative OLP, bullous OLP, and atrophic OLP.

* Indicates statistical significance at *p* < 0.05 (Mann–Whitney *U* test).

The correlation test for the OHIP‐14 values is presented in Table 4. OHIP‐14 scores showed a positive correlation with PSI (*r* = 0.438, *p* < 0.001), indicating a moderate association between poorer periodontal health and poorer OHRQoL. A weaker correlation was observed between the OHIP‐14 and the DMFT index (*r* = 0.195, *p* = 0.04), suggesting only a limited association between cumulative dental disease and its perceived impact on oral health. When stratified by OLP subtype, the association between OHIP‐14 and PSI remained consistent in both groups. However, the correlation between OHIP‐14 and DMFT was more pronounced in the reticular group and negligible in the nonreticular group. This indicates potential subgroup differences, which should be interpreted cautiously due to the exploratory nature of the analysis. There was a weak correlation between OHIP‐14 and plaque (*r* = 0.204, *p* = 0.031). No link was found between OHIP‐14 scores and dental check‐ups, PTC or bleeding gums (Table 4).

**Table 4:** Comparison of the correlations of the OHIP‐14 score with clinical form of OLP.

	Total (*n* = 112)		Reticular (*n* = 50)		Non‐reticular^a^ (*n* = 62)	
	*r* b	*p* value	*r* b	*p* value*	*r* b	*p* value
OHIP‐14—PSI	0.438	**< 0.001**	0.345	0.014*	0.413	**< 0.001**
OHIP‐14—DMFT	0.195	**0.04**	0.40	< 0.01*	−0.12	0.92
OHIP‐14—dental check‐up	−0.066	0.808	−0.094	0.517	−0.101	0.435
OHIP‐14—PTC	0.023	0.49	0.011	0.808	0.142	0.27
OHIP‐14—bleeding gums	0.152	0.11	0.82	0.57	0.139	0.282
OHIP‐14—plaque	0.204	**0.031**	0.097	0.502	0.269	**0.035**

*Note:* Bold values are statistically significant.

^a^ Erosive‐ulcerative OLP, bullous OLP, and atrophic OLP.

^b^ Spearman correlation coefficients.

Correlation tests for the PSI are described in Table 5. PSI values showed a weak correlation with DMFT (*r* = 0.190, *p* = 0.044) and a positive association with self‐reported gingival bleeding (*r* = 0.30, *p* = 0.001). This suggests that worsening periodontal status is accompanied by increased inflammatory symptoms. No relevant associations were observed between PSI and preventive dental care variables (Table 5).

**Table 5:** Comparison of the correlations of the PSI according to the clinical form of OLP.

	Total (*n* = 112)		Reticular (*n* = 50)		Non‐reticular^a^ (*n* = 62)	
	*r* b	*p* value	*r* b	*p* value	*r* b	*p* value*
**PSI—DMFT**	0.190	**0.044**	0.372	**0.008**	0.029	0.822
**PSI—dental check‐up**	0.039	0.681	−0.023	0.875	0.103	0.425
**PSI—PTC**	0.081	0.394	−0.013	0.931	0.181	0.159
**PSI—bleeding gums**	0.3	**0.001**	0.281	**0.048**	0.223	0.081
**PSI—plaque**	−0.005	0.958	0.004	0.975	−0.29	0.825

*Note:* Bold values are statistically significant.

^a^ Erosive‐ulcerative OLP, bullous OLP, and atrophic OLP.

^b^ Spearman correlation coefficients.

## 4 Discussion

Consistent with previous studies, demographic data revealed a higher prevalence of OLP in females and middle‐aged individuals. Lesions typically presented with characteristic morphology and distribution, most commonly affecting the buccal mucosa, tongue, and gingiva. Other sites were involved less frequently, with isolated or single‐site manifestations being rare (Carrozzo and Thorpe 2009; Eisen 2002).

This cross‐sectional study demonstrates that clinical subtypes of OLP are associated with differences in periodontal status. Patients with nonreticular OLP exhibited poorer periodontal conditions than those with reticular disease.

Furthermore, erosive‐ulcerative, bullous, and atrophic OLP were combined into a single nonreticular group for analytical purposes. While this approach improves comparability, it may have masked potential subtype‐specific differences and should be considered when interpreting the findings.

Periodontal diseases arise from the development of oral biofilm in dysbiosis and are influenced by the host's immune‐inflammatory response (Herrero et al. 2018). Studies indicate that the pathogenesis of both OLP and periodontitis involves biomarkers linked to inflammation‐related tissue destruction.

A recent review by Nunes et al. identified OLP as a risk factor for periodontal disease, showing more severe periodontal parameters and elevated biomarker levels in affected patients. Overall, the evidence suggests a link between OLP and the severity of periodontal disease; however, further studies with well‐defined clinical and biochemical outcomes and careful control of confounding factors are needed (Nunes et al. 2022).

Our results indicated that 61.7% of the patients had periodontitis (PSI codes 3 and 4), including 17.9% with severe periodontitis (PSI code 4). When comparing these results with data from the 5th German Oral Health Study, the figures are slightly lower than those reported for young senior women aged 65–74. According to the latter study, 71% of German women in this age group were affected by periodontitis and 18.8% presented with a severe form (PSI code 4) (Jordan et al. 2016). Overall, periodontal disease in our cohort was no more prevalent than in the general population, but comparison with population‐based datasets (e.g., the German Oral Health Study) should be interpreted cautiously due to differences in sampling frameworks, age distribution, and study design. Therefore, any similarities or differences observed should not be interpreted as direct population‐level equivalence. Instead, they should be considered only as broad contextual reference points.

There was a significant difference between the clinical forms of OLP (*p* < 0.01). Periodontitis affected 75.8% of patients in the nonreticular group. Of these patients, 24.2% suffered from a severe form of the disease. Concomitant diseases of the oral mucosa or periodontium, such as periodontitis, can exacerbate the condition or trigger the development of new lesions. In addition, painful and erosive OLP lesions often make it difficult to maintain adequate dental hygiene, resulting in patients becoming trapped in a vicious cycle. In particular, patients with erosive‐ulcerative or bullous OLP exhibit poorer periodontal parameters.

Although OLP is linked to microbiome changes and inflammation, our cohort showed no increased DMFT index, nor any differences between OLP subtypes (groups 1 and 2), indicating no overall decline in dental health.

Patients with OLP attended regular dental appointments and had an average DMFT index of 18.02, comparable to the figure reported for young seniors in the 5th German Oral Health Study (17.7) (Jordan et al. 2016). However, this comparison should be interpreted with caution as mentioned before. Accordingly, these values should not be interpreted as direct epidemiological comparisons, but rather as broad contextual reference points.

The international DMFT index is a standard measure of caries experience. As bacteria contribute to the pathogenesis of both OLP and caries, their interaction warrants further investigation (Jung and Jang 2022).

Importantly, the study did not detect any clinically meaningful associations between preventive dental care behaviors and periodontal status. This suggests that behavioral indicators alone may not adequately reflect disease burden in patients with OLP, particularly in those with symptomatic mucosal involvement.

The importance of plaque control for patients with OLP was first recognized by Erpenstein (1985) and Holmstrup et al. (1990). Although the influence of plaque on OLP has long been recognized, 24.1% of the patients examined stated not having professional tooth cleaning on a regular basis. Stone et al. (2015) demonstrated that plaque control supports OLP management by reducing symptoms and lesion severity, although it does not address the underlying cause.

However, Mergoni et al. (2019) found that improved oral hygiene did not reduce pain in OLP patients. This lack of perceived benefit may contribute to low adherence to professional cleaning, particularly as symptoms can temporarily worsen in erosive‐ulcerative or bullous forms (Mergoni et al. 2019).

Other studies (e.g., Erpenstein 1985) suggest that tooth brushing may cause minor gingival abrasions, raising concerns that existing lesions could be aggravated due to the fragile and atrophic nature of the tissue.

However, the relationship between bleeding gums, plaque, OLP severity, and oral hygiene is complex, and long‐term effects on periodontal disease could not be assessed.

When patients evaluated their own oral health, 36.6% reported bleeding gums, and 51.8% reported plaque. The significant difference between the clinical forms of gingival bleeding was striking (*p* = 0.036). Among the patients with nonreticular OLP, 45.2% experienced gingival bleeding. As Ramón‐Fluixá et al. (1999) recently demonstrated in a study of 90 patients with OLP, increased plaque and calculus deposits are associated with a significantly greater incidence of erythematous and erosive gingival lesions. Atrophic or ulcerative/erosive lesions present specific challenges, as tooth brushing can be painful, leading to plaque accumulation and negatively impacting both OLP and periodontal health (Mignogna et al. 2005).

A significant correlation was observed between PSI and gingival bleeding (*p* = 0.001), highlighting how symptom severity can interfere with oral hygiene and promote further irritation. Although the association between OLP subtypes and periodontal status is clinically relevant, it does not imply causality. Longitudinal studies are required to clarify the temporal relationships.

Nonreticular OLP is associated with greater impairment of OHRQoL than reticular OLP. Significant associations were observed between periodontal health, dental status, and quality of life; however, causal relationships cannot be inferred.

OHRQoL reflects an individual's overall well‐being in relation to oral pathology (Bennadi and Reddy 2013). Painful forms of OLP can contribute to psycho‐emotional issues. Throughout treatment, disease perception, including symptoms and psychosocial impacts, should be evaluated (Albaghli et al. 2021).

The average OHIP‐14 score of the examined patients was 13.54. Patients with erosive‐ulcerous, bullous or atrophic OLP (group 2) achieved significantly higher values (15.55) (*p* = 0.02). Parlatescu et al. (2020) reported OHIP‐14 scores of 11.77 in reticular OLP and 15.68 in atrophic, erosive, ulcerative, and bullous OLP patients.

Various studies confirm the negative impact of these forms on quality of life. Wiriyakijja et al. (2020) reported that one‐third of OLP patients experience psychological comorbidities such as anxiety or depression. Patients with ulcerative OLP had higher depression scores than those with other clinical forms of OLP (Hsu et al. 2022).

A recently published review by Yuwanati et al. (2021) concluded that OLP considerably impacts OHRQoL, irrespective of age or gender. When establishing a treatment protocol for OLP patients, clinicians should incorporate an OHRQoL assessment using PROMs such as the OHIP‐14 to monitor treatment outcomes. Oral health conditions can be triggered by psychological or physical stress, chemical irritants or chronic mechanical trauma (Yuwanati et al. 2021).

A key limitation is the heterogeneity among the included studies, particularly with regard to the clinical characterization and diagnosis of OLP. In addition, potential selection bias (e.g., the inclusion of mainly symptomatic patients) and unaccounted confounding factors such as socio‐economic, occupational, and psychological variables may have influenced the results.

The moderate association between periodontal status and OHRQoL (*p* < 0.001) highlights the clinical relevance of inflammatory oral conditions, although the cross‐sectional design precludes any causal conclusions. Previous studies showed that periodontal and chronic oral mucosal diseases negatively affect quality of life, particularly as severity increases (Ferreira et al. 2017; Ni Riordain et al. 2016).

In contrast, cumulative dental disease (DMFT) showed only weak associations with OHRQoL (*p* = 0.04), suggesting that the current inflammatory burden may be more relevant to the patient experience than historical caries exposure. Previous studies reported lower OHRQoL in patients with decayed, missing, or extensively restored teeth (Wąsacz and Chomyszyn‐Gajewska 2022).

These mechanical irritations could explain the significant correlation found between the OHIP‐14 score and the presence of plaque (*p* = 0.03). This finding suggests that good dental hygiene and effective plaque control can reduce clinical signs and improve patients’ quality of life.

A key strength of this study is the integration of clinical periodontal parameters with patient‐reported outcomes, highlighting the importance of periodontal health in OLP, particularly in symptomatic subtypes. The findings imply that periodontal inflammation can affect quality of life and that a tailored approach to plaque control can be beneficial, supporting an integrated approach to oral care despite the study limitations of the study.

### 4.1 Limitations

When interpreting the results, several limitations of this study should be acknowledged.

First, the absence of a control group without OLP limits the ability to contextualize periodontal status and OHRQoL against a non‐affected population. Therefore, the findings should be interpreted as within‐group associations rather than as disease‐specific effects.

Second, the cross‐sectional design does not permit conclusions to be drawn about temporality or causality. Consequently, it remains unclear whether periodontal deterioration contributes to OLP severity, whether symptomatic OLP impairs oral hygiene and periodontal health, or whether both are influenced by shared underlying factors.

Third, the periodontal assessment relied on the PSI, a validated screening tool that enables standardized classification, but which lacks detailed clinical parameters, thereby limiting precision. While this approach ensures feasibility in a clinical cohort, it reduces granularity. Similarly, DMFT categorization may be less sensitive to subtle differences in dental health.

Fourth, several potentially relevant confounding factors, including smoking status, systemic diseases, medication use, and socioeconomic variables, were not systematically recorded or included in multivariable analyses. Their potential influence on both periodontal outcomes and OHRQoL cannot therefore be excluded.

Fifth, the study population was derived from a single tertiary care center and consisted predominantly of female patients. This may limit the generalizability of the findings to broader or more balanced populations.

Sixth, erosive‐ulcerative, bullous, and atrophic OLP were combined into a single nonreticular group for analytical purposes. While this approach improves statistical feasibility and reflects shared clinical characteristics, it may have masked potential subtype‐specific differences which should be considered when interpreting the results.

## 5 Conclusions

Within the limitations of this cross‐sectional study, different clinical subtypes of OLP are associated with differences in periodontal status and oral health‐related quality of life. Nonreticular OLP is associated with poorer periodontal conditions and greater impairment of OHRQoL. Periodontal status demonstrated a moderate association with OHRQoL, whereas the DMFT index showed only weak associations.

However, these findings should be interpreted as exploratory associations rather than definitive clinical relationships, particularly given the observational study design and the absence of multivariable adjustment. Furthermore, the grouping of erosive‐ulcerative, bullous, and atrophic OLP into a combined nonreticular category may have masked potential subtype‐specific differences.

Longitudinal and controlled studies are required to determine temporal relationships, underlying mechanisms and potential confounding factors. Additional research is needed to determine whether OLP is a risk factor for the progression and worsening of periodontal disease or a coincidental finding.

## Author Contributions

Conceptualization: Linda Daume, Ole Oelerich, Johannes Kleinheinz, Sonja Sielker, and Lauren Bohner. Methodology: Linda Daume. Validation: Johannes Kleinheinz and Lauren Bohner. Formal analysis: Lauren Bohner. Investigation: Linda Daume and Ole Oelerich. Resources: Linda Daume. Data curation: Linda Daume and Ole Oelerich. Writing—original draft preparation: Linda Daume. Writing—review and editing: Lauren Bohner and Johannes Kleinheinz. Visualization: Linda Daume. Supervision: Johannes Kleinheinz and Lauren Bohner. Project administration: Linda Daume. All authors have read and agreed to the published version of the manuscript.

## Ethics Statement

The study was approved by the Ethics Committee of the Medical Association of Westphalia‐Lippe and the Westphalian Wilhelms University Münster (Ref. No. 2019‐033.f‐S). All experiments were performed in accordance with the Declaration of Helsinki.

## Consent

Informed consent was obtained from all the subjects.

## Conflicts of Interest

The authors declare no conflicts of interest.

## Data Availability

The datasets used and analyzed during the current study are available from the corresponding author on reasonable request.
